# The Pharmaceutical Industry in 2024: An Analysis of the FDA Drug Approvals from the Perspective of Molecules

**DOI:** 10.3390/molecules30030482

**Published:** 2025-01-22

**Authors:** Beatriz G. de la Torre, Fernando Albericio

**Affiliations:** 1 School of Laboratory Medicine and Medical Sciences, College of Health Sciences, University of KwaZulu-Natal, Durban 4001, South Africa; 2 School of Chemistry and Physics, University of KwaZulu-Natal, Durban 4001, South Africa; 3 Department of Organic Chemistry, University of Barcelona, 08028 Barcelona, Spain

**Keywords:** antibodies, biologics, chemical entities, deuterated drugs, fluorine-based drugs, imaging, natural products, new chemical entities, oligonucleotides, PFAS, peptides, polyfluoroalkyl substances, TIDES

## Abstract

The U.S. Food and Drug Administration (FDA) has authorized 50 new drugs in 2024, which matches the average figure for recent years (2018–2023). The approval of 13 monoclonal antibodies (mAbs) sets a new record, with these molecules accounting for more than 25% of all drugs authorized this year. Three proteins have been added to the list of biologics, and with the inclusion of four TIDES (two oligonucleotides and two peptides), only one in three approved drugs this year is a small molecule. As of 2023, no antibody-drug conjugates (ADCs) have reached the market this year. Two deuterated drugs have been approved, bringing the total approvals for this class of compounds to four. This year saw the authorization of two more PEGylated drugs—both peptides—highlighting a renewed interest in this strategy for extending drug half-life, despite the setback caused by the withdrawal of peginesatide from the market in 2014 due to adverse side effects. *N*-aromatic heterocycles and fluorine atoms are present in two-thirds of all the small molecules approved this year. Herein, the 50 new drugs authorized by the FDA in 2024 are analyzed exclusively on the basis of their chemical structure. They are classified as the following: biologics (antibodies, proteins), TIDES (oligonucleotides and peptides), combined drugs, natural products, F-containing molecules, nitrogen aromatic heterocycles, aromatic compounds, and other small molecules.

## 1. Analysis

**Figure 1:**
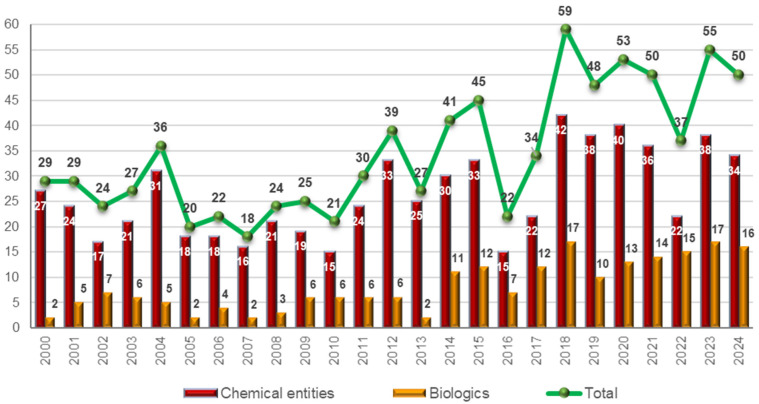
Drugs (new chemical entities and biologics) approved by the FDA in the last 25 years. Adapted with permission from ref. [2]. Copyright 2024, copyright MDPI [1,2].

During 2024, the U.S. Food and Drug Administration (FDA) has approved 50 new drugs [1]. This figure matches the average of 50 per year for the period 2018–2023 (Figure 1), reaffirming that the COVID-19 pandemic has not significantly impacted the pharmaceutical industry. On the contrary, analysts believe that the industry’s rapid response in developing vaccines has helped boost public confidence in the pharmaceutical sector.

Closer inspection of the 50 drugs approved in 2024 by type of molecule reveals 16 biologics (17, 10, 13, 14, 15, and 17 in 2018, 2019, 2020, 2021, 2022, and 2023, respectively), 4 TIDES (2 oligonucleotides and 2 peptides in 2024 vs. 3 and 1 in 2018, 2 and 3 in 2019 and 2020, 2 and 8 in 2021, 1 and 4 in 2022, and 4 and 5 in 2023), and 30 so-called small molecules, bringing the total to 34 new chemical entities (NCEs), including TIDES and small molecules (Figure 1) [1,2].

In 2024, the proportion of biologics among NCEs was slightly higher than in previous years: 32% of all drug approvals have been for biologics, compared to 28% over the past six years. This increase reflects the growing importance of biologics in the pharmaceutical market. Indeed, out of a total of 304 drugs approved over these six years by the FDA, 87 are biologicals [2].

This year, the Center for Biologics Evaluation and Research (CBER) has given the go-ahead for 24 new Biologics License Applications, a number similar to the 22 approvals in 2023, which at that time marked a notable increase compared to the 12 and 13 registered in 2022 and 2021, respectively [3,4]. The Biologics License Applications authorized this year included several medical devices and computer software related to blood management and analysis and also diagnostic tests. 

As in previous years, this manuscript analyzes the 50 new drugs approved by the FDA in 2024 based solely on their chemical structure. The number of references has been minimized to ensure a smooth flow of the manuscript. Ref. [1] provides more information.

## 2. Discussion

This year, the FDA has approved 16 biologics (Table 1), confirming the trend observed in recent years. In this regard, since 2015, the number of biologics approved each year has consistently been in the double digits, except in 2016, when only 7 were authorized (7). Of the biologics given the green light in 2024, 13 are monoclonal antibodies (mAbs), surpassing the 12 approved last year and setting a new record for this class of drugs. The remaining approvals have been given to three proteins.

Once again, mAbs are the most approved drug class, not only among biologics but across all drugs, accounting for 25% of all medications authorized. Although cancer continues to be the principal target for mAbs (50% of the total), the number of medical indications is widening through the incorporation of Alzheimer’s disease and skin and blood conditions, among others. Two of the mAbs to receive the green light this year, concizumab-mtci (Alhemo^TM^) and marstacimab-hncq (Hympavzi^TM^), are indicated for the same target, namely the prevention or reduction of bleeding episodes related to hemophilia A or B. Four of the mAbs are indicated for the treatment of skin-related disorders: lebrikizumab-lbkz (Ebglyss^TM^) for eczema; nemolizumab-ilto (Nemluvio^TM^) for hard, raised bumps; and cosibelimab-ipdl (Unloxcyt^TM^) for cutaneous squamous cell carcinoma. If proteins are considered, a fourth biologic, letibotulinumtoxinA-wlbg (Letybo^TM^), has also been approved for the same target. Therefore, a quarter of the mAbs authorized this year are indicated for the treatment of the skin.

This year, a third mAb, namely donanemab-azbt (Kisunla^TM^), has been given the green light for the treatment of Alzheimer’s disease, and it was preceded by approvals for aducanumab-avwa (Aduhelm^TM^) in 2021 and lecanemab (Leqembi^TM^) in 2023. The approval of aducanumab-avwa raised serious discussion regarding its effectiveness. While the latter two were developed by Biogen, Eli Lilly is behind donanemab-azbt. This drug is specially indicated for the treatment of individuals with early symptomatic Alzheimer’s disease, including mild cognitive impairment or mild dementia. As of 2023, no antibody-drug conjugates (ADCs) have been approved this year. Thus, 14 ADCs have been authorized to date, the last one in 2022.

This year, three protein-based drugs have been given the green light by the FDA. Interestingly, Anktiva^TM^ is a fixed-dose combination of an interleukin-15 receptor agonist with Bacillus Calmette-Guérin for the treatment of bladder cancer. The first is a fusion protein that contains a human IL-15 variant (nogapendekin alfa) attached to a dimeric human IL-15Rα sushi domain/human IgG1 Fc (inbakicept).

Despite being one of nature’s most dangerous toxins, botulinum toxin has proven to be a rich source of new drugs, predominantly targeting facial wrinkles and glabellar lines, being referred to as “Botulinum toxin: bioweapon & magic drug” [5]. In this context, this year letibotulinumtoxinA-wlbg (Letybo^TM^) has joined onabotulinumtoxinA (Botox^TM^), abobotulinumtoxinA (Dysport^TM^/Azzalure^TM^), rimabotulinumtoxinB (Myobloc^TM^), incobotulinumtoxinA (Xeomin^TM^/Bocouture^TM^), and prabotulinumtoxinA (Jeuveau^TM^), which were already on the market. Finally, among the proteins, sotatercept-csrk (Winrevair^TM^) has been approved for the treatment of pulmonary arterial hypertension. This drug contains the extracellular domain of the activin type 2 receptor, expressed as a recombinant fusion protein with an immunoglobulin Fc domain.

Oligonucleotides and peptides, collectively known as TIDES, are classified as chemical entities, meaning that regulatory agencies require their approval based on criteria similar to those for small molecules. However, it should be noted that, in many cases, the structural complexity of TIDES is more similar to that of biologics than to that of small molecules.

Although TIDES are typically more active than small molecules, they require smaller doses of the Active Pharmaceutical Ingredient (API). However, in some cases, such as peptides designed for diabetes and obesity, which have more than 30 residues and a large market potential, their production poses significant challenges in both upstream and downstream processes. TIDES have transcended the scientific field and gained significant visibility in society, with a notable presence in the mass media. While five peptides and four oligonucleotides were released into the market in 2023, two peptides and one oligonucleotide have been approved by the FDA this year. The two peptides, palopegteriparatide (Yorvipath^TM^) and pegulicianine (Lumisight^TM^) contain a large polydisperse PEG moiety as indicated in the name of the API. The authorization of these medications reflects the resurgence of pegylated drugs after the withdrawal of peginesatide (Omontys^TM^), a hyperpegylated disulfide cyclic peptide dimer, from the market in 2013. In this context, 2021 saw the approval of another highly pegylated peptide, namely pegcetacoplan (Empaveli^TM^), while zilucoplan (Zilbrysq^TM^), which also contains a defined large PEG, was authorized in 2023.

**Figure 2:**
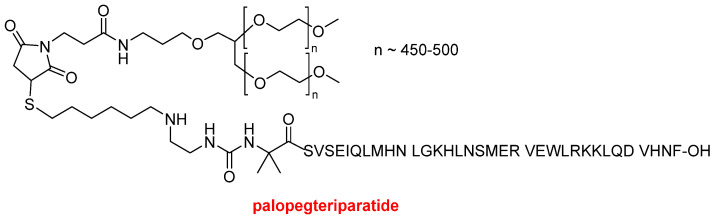
Structure of palopegteriparatide.

Palopegteriparatide (Yorvipath^TM)^ is a hormone replacement therapy indicated for the treatment of hypoparathyroidism. It contains fragment 1–34 of the human parathyroid hormone (PTH), which is also the API of teriparatide (Forteo^TM^), a drug approved by the FDA in 2002 for the treatment of osteoporosis. In palopegteriparatide, PTH (1-34) ends in an Aib residue and is conjugated to a branched methoxypolyethyleneglycol (mPEG) carrier by a proprietary TransCon Linker based on the maleimide moiety (Figure 2).

**Figure 3:**
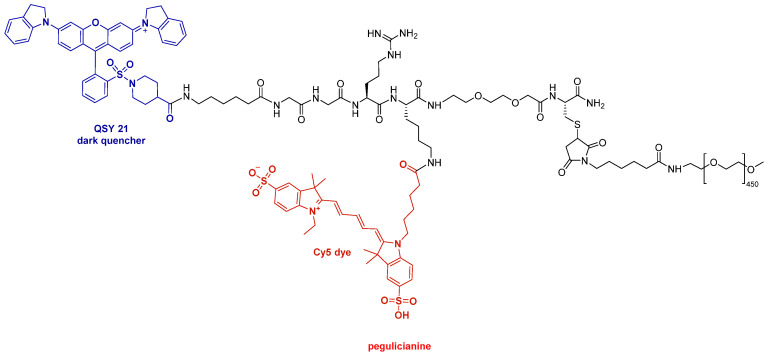
Structure of pegulicianine.

The second peptide approved this year is an optical imaging agent for the detection of cancerous tissues. In this regard, pegulicianine (Lumisight^TM^) is a clear example that, in addition to their biological properties, peptides are excellent scaffolds for building complex multivalent molecules (Figure 3). The peptide linear chain of pegulicianine Ahx-Gly-Gly-Arg-Lys-AEEA-Cys-NH_2_ (Ahx for 6-aminohexanoic, AEEA for [2-[2-aminoethoxy]ethoxy]acetic acid), where the amino of the Ahx is linked to a dark quencher (QSY21), the amino side chain of the Lys to the fluorophore (Cy5), and the thiol of Cys to a polydisperse mPEG, which is responsible for the solubility. Pegulicianine is a prodrug that is activated by metalloproteinases and cathepsins B, K, L, and S, which are overexpressed in many tumors and whose cleavage site is Gly-Arg.

**Figure 4:**
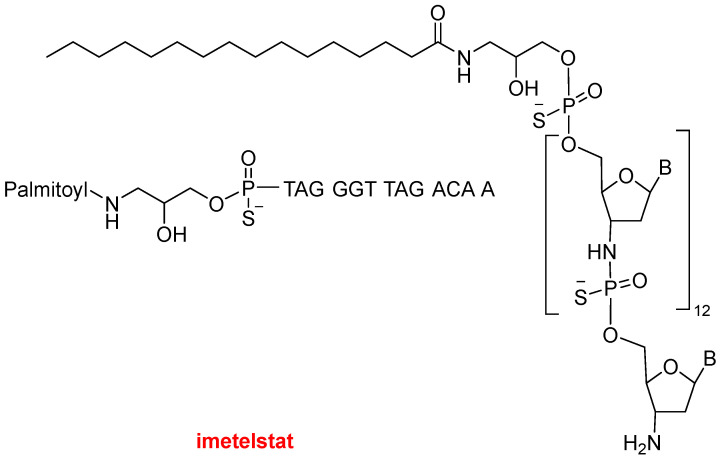
Structure of imetelstat.

This year the FDA has authorized two oligonucleotides, bringing the total number of approvals for these molecules to 21 to date. Imetelstat (Rytelo^TM^), a DNA analog, is a human telomerase inhibitor indicated for the treatment of myelodysplastic syndromes (hematologic neoplasia). Imetelstat presents a thiophosphoramidate structure conjugated to a palmitoyl moiety (Figure 4).

**Figure 5:**
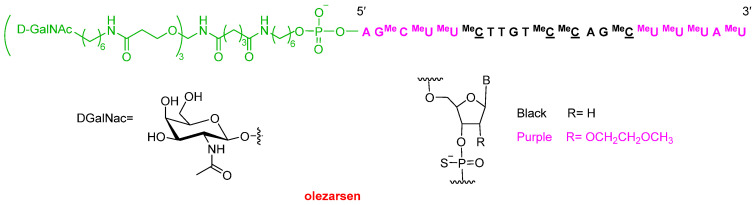
Structure of olezarsen.

Olezarsen (Tryngolza^TM^) has been given the green light for the treatment of familial chylomicronemia syndrome, which occurs when the body does not break down lipids properly. From a structural perspective, olezarsen is an antisense oligonucleotide containing the dendrimer of *N*-acetyl galactosamine (GalNac) [Enhanced Stabilization Chemistry (ESC)], which assures the delivery of the drug to hepatocytes. It contains 19 thiophosphate linkages (all but the one that links it to the ESC system) and is formed by 20 phosphorothioate (10 metoxyethoxy situated at both ends and 10 deoxy) nucleotides (Figure 5). This ESC system is common in the latest oligonucleotides to have reached the market.

Like last year, in 2024, five drugs containing more than one API have been approved by the FDA. In addition to Anktiva^TM^, which contains nogapendekin alfa and inbakicept, four drugs are classified as combination drugs.

**Figure 6:**
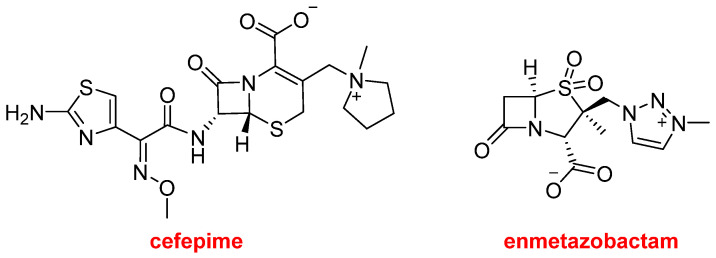
Structure of cefepime and enmetazobactam, both components of Exblifep^TM^.

Exblifep^TM^ is a fixed-dose combination that contains cefepime and enmetazobactam and is indicated for the treatment of individuals with complicated urinary tract infections (Figure 6). The first component is a fourth-generation cephalosporin antibiotic with broad-spectrum activity against Gram-positive and Gram-negative bacteria. Cefepime was approved thirty years ago and is now marketed as a generic drug. Enmetazobactam is a β-lactamase inhibitor with a unique mechanism of action. Exblifep^TM^ is as potent as meropenem. This antibiotic-β-lactamase inhibitor combination is the basis of several new drugs, including Xacduro^TM^, containing sulbactam (β-lactam antibacterial and β-lactamase inhibitor) and durlobactam (β-lactamase inhibitor), approved last year.

**Figure 7:**
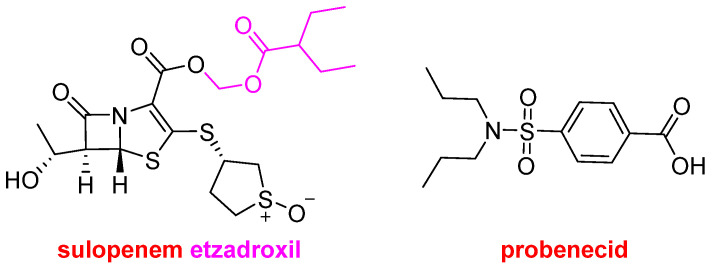
Structure of sulopenem etzadroxil and probenecid, both components of Orlynvah^TM^.

Orlynvah^TM^ contains sulopenem etzadroxil and probenecid (Figure 7) as a fixed-dose drug, and, like Exblifep^TM^, it is also indicated for the treatment of urinary tract infections. Sulopenem etzadroxil, the prodrug of sulopenem, which is an antibacterial that was developed in Japan towards the end of the last century, reached clinical phase III but never was approved. Probenecid is a renal tubular transport inhibitor that is already commercialized as Probalan^TM^. Probenecid is a sulfonamide indicated for the treatment of gout and hyperuricemia, and it acts by increasing uric acid excretion in urine.

**Figure 8:**
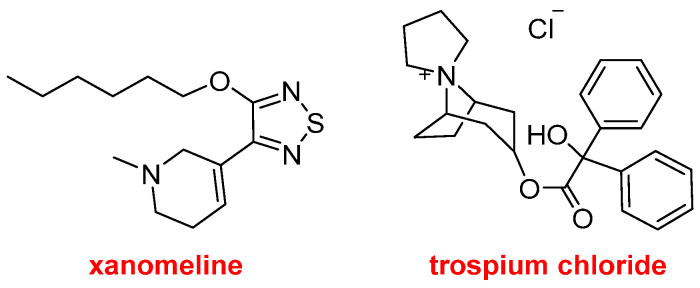
Structure of xanomeline and trospium chloride, both components of Cobenfy^TM^.

Cobenfy^TM^, which has been approved for the treatment of schizophrenia, contains xanomeline and trospium chloride (Figure 8). The first compound is a muscarinic M4 and M1 receptor agonist, while the second one is a non-selective muscarinic antagonist. The mechanism of action exerted by Cobenfy^TM^ is not fully understood, but it appears that trospium chloride counteracts some of the side effects of xanomeline in peripheral tissues.

**Figure 9:**
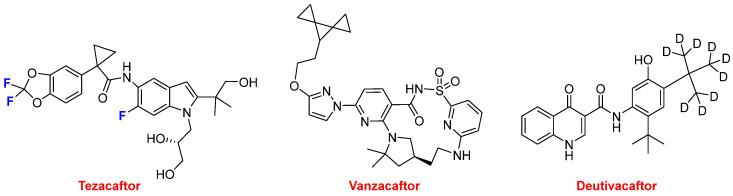
Structure of tezacaftor, vanzacaftor, and deutivacaftor, components of Alyftrek^TM^.

Alyftrek^TM^ is a fixed-dose combination drug containing tezacaftor, vanzacaftor, and deutivacaftor and is indicated for the treatment of cystic fibrosis (Figure 9). Tezacaftor and vanzacaftor bind to different sites on the cystic fibrosis transmembrane conductance regulator (CFTR) protein and have an additive effect in increasing the amount of CFTR protein delivered to the cell surface. Deutivacaftor, which is a poly-deuterated compound (see below), facilitates the channel gating of the CFTR protein at the cell surface. Tezacaftor forms part of two fixed-dose combination drugs approved for the same target in previous years, namely ivacaftor (Symdeko^TM^) authorized in 2018 and ivacaftor and elexacaftor (Trikafta^TM^) in 2021.

With the five fixed-dose combination drugs given the green light this year, the FDA has approved a total of 27 such drugs between 2016 and 2024. These combination drugs involve not only small molecules but also proteins, such as Anktiva^TM^, which has been approved this year, and mAbs, such as Opdualag^TM^, which was authorized in 2022. This drug discovery strategy often repurposes drugs that have already been accepted by regulatory agencies.

**Figure 10:**
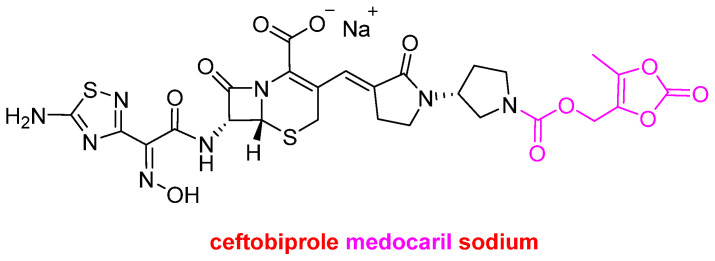
Structure of ceftobiprole medocaril sodium.

Natural products continue to serve as a vital starting point for the development of modern drugs. In addition to biologics, TIDES, cefepime, enmetazobactam, and sulopenem etzadroxil are presented in fixed-dose combination drugs; another drug draws inspiration from the natural product ecosystem. This is yet another cephalosporin-based drug, namely Zevtera^TM^ (ceftobiprole medocaril sodium) (Figure 10). This is a prodrug of ceftobiprole, which is a fifth-generation cephalosporin antibacterial. Ceftobiprole medocaril is converted rapidly and almost completely to the active drug, ceftobiprole, upon infusion by type A esterases [6]. Zevtera^TM^ is indicated for three uses: treatment of bloodstream infections, bacterial infections of the skin and associated tissue, and community-acquired bacterial pneumonia.

**Figure 11:**
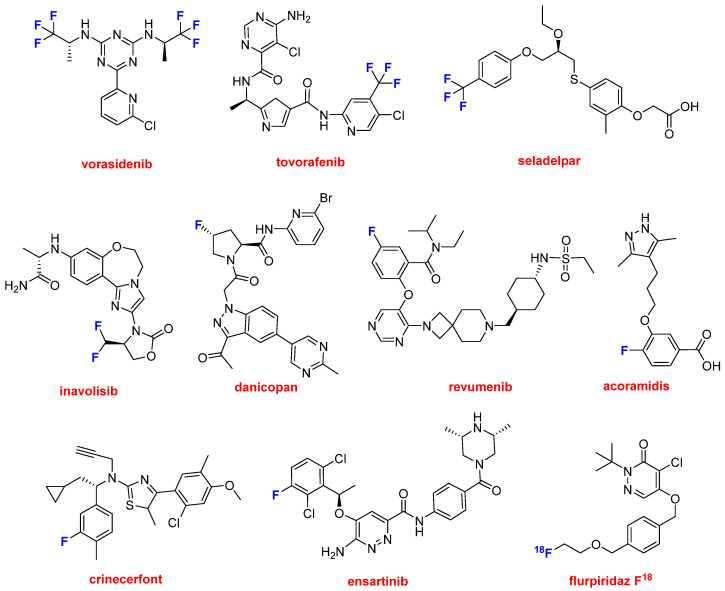
Structure of F-containing drugs.

Once again, we must highlight the role of fluorine atoms in drug discovery. This year, eleven drugs contain one or more F (Figure 11) atoms, accounting for 22% (11 out of 50) of all the drugs authorized in this period [7]. Excluding biologics, this figure increases to 32% (11 out of 34), meaning that one-third of all chemical entity-based drugs contain F.

Tezacaftor, as part of Alyftrek^TM^, contains a CF_2_ moiety and one F atom. Three drugs contain CF_3_ moieties. Vorasidenib (Voranigo^TM^), which is a 1,3,5-triazine holding two CF_3_, and pyridine. It has been approved for the treatment of grade 2 astrocytoma (growth of astrocytes, which are cells that support and connect nerve cells in the brain and spinal cord) and oligodendroglioma (brain tumor from glial cells or oligodendrocytes). Vorasidenib inhibits isocitrate dehydrogenase-1 and 2 (IDH1 and IDH2) enzymes.

Two drugs approved this year, tovorafenib (Ojemda^TM^) and seladelpar (Livdelzi^TM^), contain one CF_3_ moiety. The former is a kinase inhibitor indicated for the treatment of refractory or relapsed pediatric low-grade gliomas. It also contains pyrimidine, thiazole, and pyridine moieties, forming an extended structure. Seladelpar is indicated to treat primary biliary cholangitis (inflammation of the bile duct system) and is presented as a lysine dihydrate salt. Its mode of action is not fully understood. Inavolisib (Itovebi^TM^), a kinase inhibitor that also contains a CF_2_ moiety, similar to tezacaftor in Alyftrek^TM^, has been approved for the treatment of metastatic breast cancer. It shows an extended structure with oxo-oxazolidine, imidazole, and benzoxazepine moieties.

This year has seen the approval of six drugs containing one F atom. Danicopan (Voydeya^TM^) is a complement factor D inhibitor indicated to treat extravascular hemolysis with paroxysmal nocturnal hemoglobinuria. Its extended structure contains pyrimidine, indazole, and pyridine moieties. Revumenib (Revuforj^TM^), authorized for the treatment of refractory or relapsed acute leukemia, is a menin inhibitor. It also shows an extended structure and contains a pyrimidine moiety. Acoramidis (Attruby^TM^) has been given the green light for the treatment of cardiomyopathy. It is a selective stabilizer of transthyretin and contains a pyrazole structure. Crinecerfont (Crenessity^TM^) is a selective corticotropin-releasing factor type 1 receptor antagonist indicated for the treatment of congenital adrenal hyperplasia. It contains a thiazole structure. Ensartinib (Ensacove^TM^) is a kinase inhibitor of anaplastic lymphoma kinase, and it also inhibits other kinases. It contains a pyridazine moiety and is indicated for the treatment of non-small cell lung cancer.

Flurpiridaz F 18 (Flyrcado^TM^) is the only radioactive diagnostic drug approved this year, specifically for evaluating myocardial ischemia and infarction using positron emission tomography (PET). Flurpiridaz F 18 is a simplified analog of pyridaben, which is an inhibitor of mitochondrial complex 1. Both contain a pyridazin-3-one moiety.

**Figure 12:**
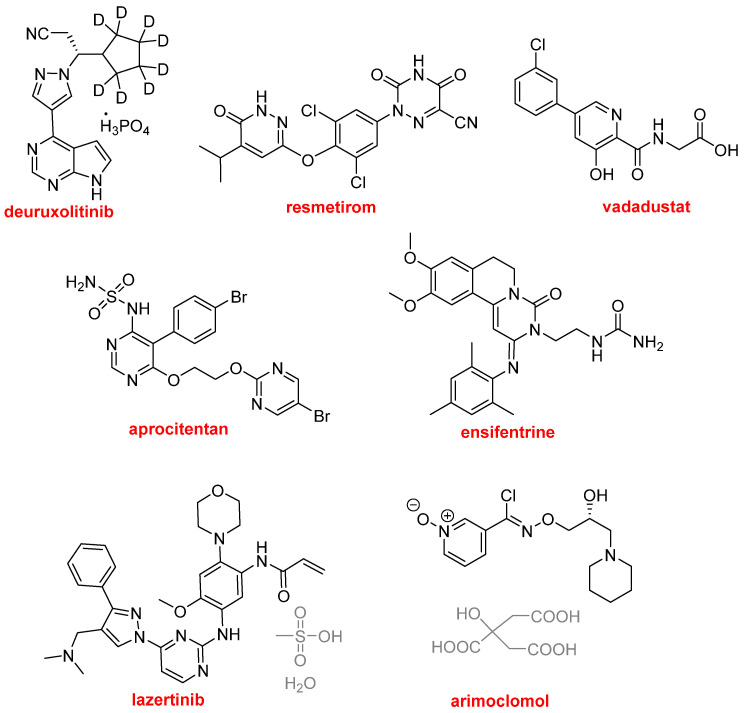
Structures of drugs containing *N*-aromatic heterocycles.

*N*-aromatic moieties are also a constant in all drugs on the market. This year has not been an exception to this rule. Thus, ten out of eleven F-containing drugs, as well as cefepime and enmetazobactam present in Exblifep^TM^, and vanzacaftor present in Alyftrek^TM^, and ceftobiprole medocaril sodium, also contain this kind of heterocycle. In addition to these, eight more drugs belong to this class (Figure 12), representing a total of 22 out of 50 drugs (44% of all drugs, or 73% when biologics and TIDES are excluded).

Deuruxolitinib, sold under the brand name Leqselvi^TM^, is indicated for the treatment of alopecia areata. It is a Janus kinase inhibitor, but its use is accompanied by a serious warning due to the potential increase in malignancies, cardiovascular death, myocardial infarction, stroke, and thrombosis. It contains pyrazole and pyrrolopyrimidine structures. The most notable feature of this drug is the polydeuteration of a cyclopentyl moiety. This is the second drug this year to present this characteristic. Deutivacaftor, as part of Alyftrek^TM^, contains a deuterated tert-butyl group. In 2017 and 2022, deutetrabenazin (Austedo^TM^), which contains two deuterated methyl groups, and deucravacitinib (Sotyktu^TM^), with one deuterated methyl group, were the first two deuterated drugs to be approved by the FDA, respectively. Resmetirom (Rezdiffra^TM^) has been approved for the treatment of noncirrhotic non-alcoholic steatohepatitis (NASH). It is a partial agonist of the thyroid hormone receptor beta and contains pyridazine and triazine moieties. Vadadustat (Vafseo^TM^) is a reversible inhibitor of HIF-prolyl-4-hydroxylases indicated for the treatment of anemia caused by chronic kidney disease. It contains a pyridine structure. Aprocitentan (Tryvio^TM^) is an endothelin receptor antagonist used for the treatment of high blood pressure. It contains two pyrimidine moieties and one sulfamide moiety. Mavorixafor (Xolremdi^TM^) has been approved for the treatment of warts, hypogammaglobulinemia, infections, and myelokathexis (WHIM) syndrome. It is a CXC chemokine receptor 4 antagonist, and it contains benzimidazole and tetrahydroquinoline moieties. Ensifentrine (Ohtuvayre^TM^) has been authorized for the treatment of chronic obstructive pulmonary disease (COPD). It inhibits phosphodiesterases 3 and 4. Lazertinib (Lazcluze^TM^) is a kinase inhibitor that has been approved for the treatment of non-small cell lung cancer. It contains pyrazole and pyrimidine structures. Arimoclomol (Miplyffa^TM^) has been given the green light for the treatment of Niemann-Pick disease type C (inability of the body to transport lipids inside cells, therefore leading to lipid accumulation in tissues). It is a pyridine *N*-oxide.

**Figure 13:**
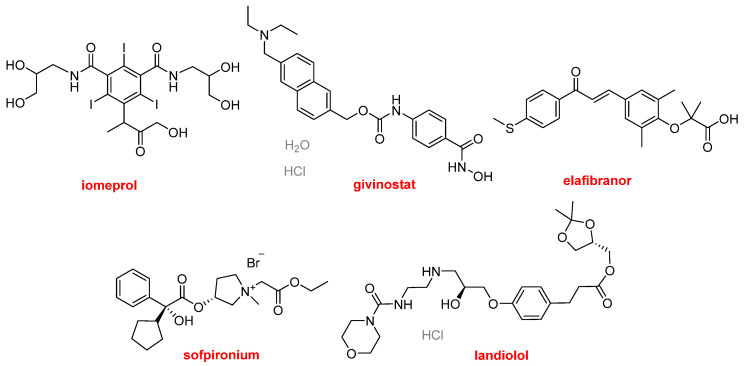
Structures of APIs containing aromatic rings.

This year, five drugs with aromatic moieties have been approved (Figure 13). Iomeprol (Iomervu^TM^) is used as a contrast for imaging, including cerebral, coronary, visceral, and peripheral arteriography, and computed tomography (CT) of the head and body. It is a 2,4,6-triiodo derivative with almost 50% of iodine content. Givinostat (Duvyzat^TM^), a hydroxamic acid, is indicated to treat Duchenne muscular dystrophy. It is a histone deacetylase (HDAC) inhibitor and, although it reduces inflammatory processes and muscle loss, its precise mechanism of action is still not fully understood. Elafibranor (Iqirvo^TM^) has been authorized for the treatment of primary biliary cholangitis in combination with ursodeoxycholic acid. Elafibranor and its main metabolite (GFT1007) are peroxisome proliferator-activated receptor (PPAR) agonists. However, the mechanism of action is not well understood. Elafibranor is also being studied for the treatment of endocrine and metabolic diseases, including type 2 diabetes, dyslipidemia, and metabolic dysfunction-associated steatohepatitis (MASH). Sofpironium (Sofdra^TM^) has been approved for the treatment of primary axillary hyperhidrosis (excessive sweating). It is a competitive inhibitor of acetylcholine receptors located on certain peripheral tissues, including sweat glands. The fifth aromatic moiety to receive the green light this year is landiolol (Rapiblyk^TM^), which is indicated for the treatment of supraventricular tachycardia. It is a beta-1-adrenoreceptor antagonist that inhibits the positive chronotropic effects of the catecholamines epinephrine and norepinephrine on the heart, where these receptors are located.

**Figure 14:**
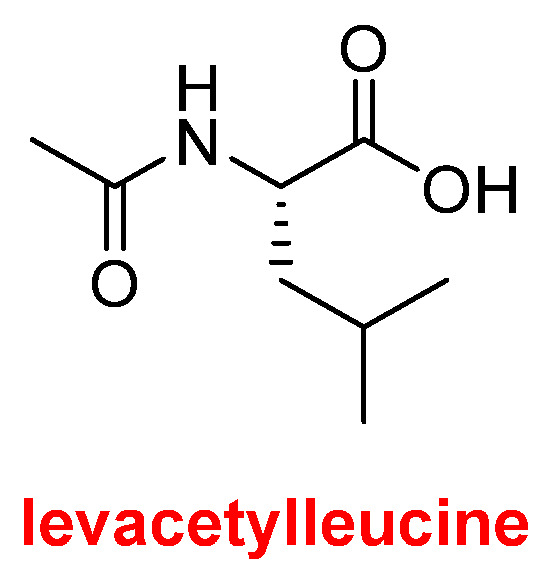
Structures of levacetylleucine.

Levacetylleucine (Aqneursa^TM^) (Figure 14), which is acetyl-L-leucine, has been approved for the treatment of neurological manifestations of Niemann-Pick disease type C (inability of the body to transport lipids inside cells, thus leading to lipid accumulation in tissues) similar to Miplyffa^TM^ discussed above. Its mechanism of action is unknown. 

**Figure 15:**
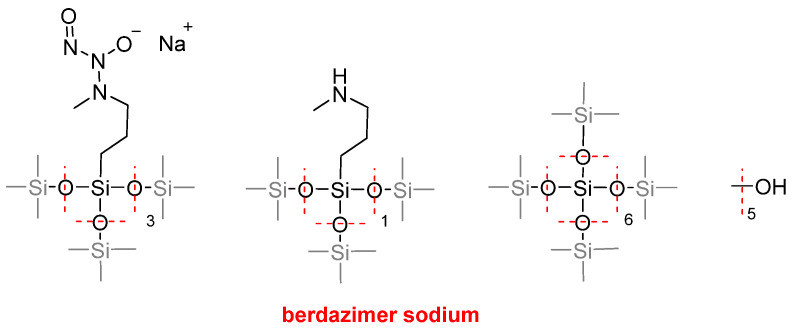
Structures of berdazimer sodium.

Berdazimer sodium (Zelsuvmi^TM^) (Figure 15) is a partially hydrolyzed co-silicate composed of two silasesquioxanes: one with 3-(methylamino)propyl moieties and the other with 3-(1-methyl-2-nitroso-2-oxidohydrazin-1-ylyl)propyl moieties. It is indicated for the topical treatment of molluscum contagiosum. It is a nitric oxide-releasing agent with an unknown mechanism of action.

## 3. Conclusions and Perspectives

In recent years, we have started this section of our annual article referring to COVID. As we anticipated last year, COVID is no longer ranked as a global concern in 2024, as it is now understood by society and the medical profession as a “new flu”. It is likely that people will receive a COVID vaccination annually, alongside the flu vaccine. However, we must consider the emergence of another disease related to COVID, namely long COVID. In most cases, it is a chronic condition, with symptoms that appear and disappear cyclically. It can be mild or severe, and, in some cases, it can lead to disability. The number of new cases of long-term COVID is decreasing, as it is directly related to the presence of COVID, which is receding. Long COVID must be treated as a new disease, and targeted treatments must be developed and investigated.

This year, no drug from the so-called GLP-1 class has been approved, but this class of drugs remains one of the pharmaceutical industry’s driving forces. In this regard, the approval of retratutide is eagerly anticipated, followed by that of cagrilintide, mazdutide, pemvidutide, and survodutide (in alphabetical order), with more to come. Likewise, semaglutide (GLP-1 agonist) and tirzepatide [double agonist (GLP-1 and GIP) have been authorized for indications other than diabetes and obesity. In this regard, semaglutide has been authorized for reducing the risk of cardiovascular disease in individuals presenting a certain degree of obesity, while tirzepatide has very recently received the green light for the treatment of severe obstructive sleep apnea. This is an indication that a priori is very far from the original one. Finally, both drugs are being studied for other indications, including anxiety, alcoholism disorder, depression, and kidney disease. The introduction of these drugs for the treatment of mental and even neurodegenerative illnesses will elevate their status from “blockbusters” to “panaceas”.

In terms of sustainability, the chemical-pharmaceutical industry faces a significant dilemma. On the one hand, there is a pressing need to eliminate the use and production of per- and polyfluoroalkyl substances (PFAS) due to their harmful effects on human health. The C-F bond is extremely strong in organic chemistry, making it highly resistant to degradation, which results in the contamination of soil and water (ground and surface). It is anticipated that the use of trifluoroacetic acid (TFA) will be restricted in Europe [8]. On the other hand, nearly 40% of chemically synthesized drugs contain F, and their production relies on PFAS. It is clear that these drugs are highly beneficial for public health and that F is irreplaceable—substituting it with H in the same compound would nullify its biological activity. However, the production of these drugs necessitates the use of environmentally harmful substances.

**Figure 16:**
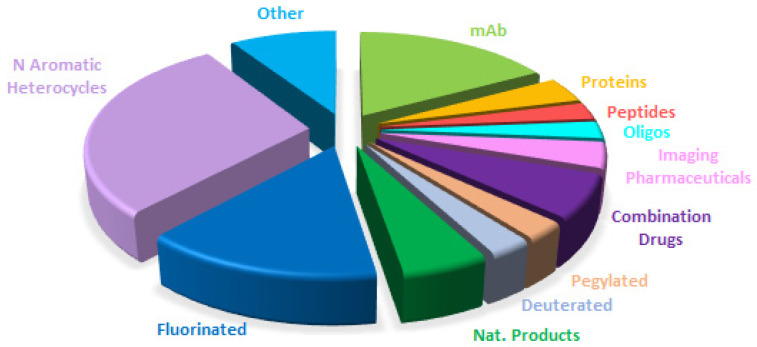
Drugs approved by the FDA in 2024 are classified on the basis of chemical structure (drugs can belong to more than one class). Adapted with permission from ref. [1]. Copyright 2024, copyright MDPI.

Three antibiotics have been approved this year; two are fixed-dose combination drugs (Figure 16), but all of them have structures (cephalosporin and penem derivatives) that have been known for more than twenty years, thereby indicating the difficulties encountered in bringing de novo antibiotics into the market.

2024 has been an excellent year in terms of the number of new drugs approved (50), consistent with the figures in recent years [55 (2023), 37 (2022), 50 (2021), 53 (2020), 48 (2019), and 59 (2018)], thereby confirming the robustness of the pharmaceutical industry. This year, 16 biologics have been authorized, matching the average of the last six years, which totaled 86 approvals. With 13 approvals this year—one more than each of the previous two years—mAbs have consolidated their status as the most significant class of drugs. Although cancer continues to be the most important indication for mAbs, this year three have been authorized for skin targets. Furthermore, the third mAb for Alzheimer’s disease, namely donanemab-azbt, has received the green light, following lecanemab in 2023 and aducanumab-avwa in 2021.

TIDES—two oligonucleotides and two peptides—have joined the ranks of this class of drugs. While there have been fewer authorizations than last year (9), peptides and oligonucleotides still represent almost 10% of all drugs approved.

TIDES and biologics together account for 40% of all drugs approved this year (20 over 50), reaffirming the slight decrease in the so-called small molecules in the pharmaceutical market, which predominated just a few years ago.

The two peptides approved this year, palopegteriparatide (Yorvipath^TM^) and pegulicianine (Lumisight^TM^), contain large polydisperse PEG. In comparison, three pegylated drugs received the green light in 2023: two TIDES—the peptide zilucoplan (Zilbrysq^TM^) and the oligonucleotide avacincaptad pegol (Izervay^TM^)—and the enzyme pegunigalsidase alfa (Elfabrio^TM^). This trend reaffirms the resurgence of PEG as a component of drugs, despite the issues associated with peginesatide (Hematide^TM^, Omontys^TM^), which was withdrawn from the market in 2014.

In 2024, two deuterated drugs have been approved, bringing the total number of these drugs to have been granted FDA approval to four. These deuterated drugs show a longer half-life, attributed to reduced metabolic rates resulting from the kinetic isotope effect. It will be interesting to observe whether this class of drugs becomes consolidated in the coming years.

This year, four FDA-approved drugs have been derived from natural products. Three of these—cefepime, enmetazobactam, and sulopenem etzadroxil—are components of fixed-dose combination drugs, while the fourth is Zevtera^TM^ (ceftobiprole medocaril sodium). All of these drugs are anti-infectives, featuring cephalosporin and penem structures that have been well-established in the pharmaceutical field for many years.

Three drugs have been approved as imaging agents: the radioactive flurpiridaz F 18 (Flyrcado^TM^), iomeprol (Iomervu^TM^), and pegulicianine (Lumisight^TM^), which is built on a peptide scaffold.

If we focus solely on the so-called small molecules, excluding biologics and TIDES, it seems that drug design often involves combining *N*-aromatic heterocycles with the presence of F. This year has followed this trend, with 23 out of 30 drugs (77%) containing either *N*-aromatic heterocycles or F.

**Figure 17:**
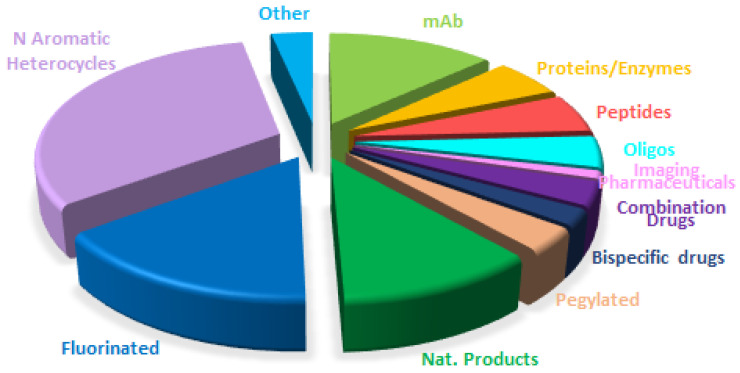
Similar to Figure 16 for 2023, taken with permission from ref. [2]. Copyright 2024, copyright MDPI.

**Figure 18:**
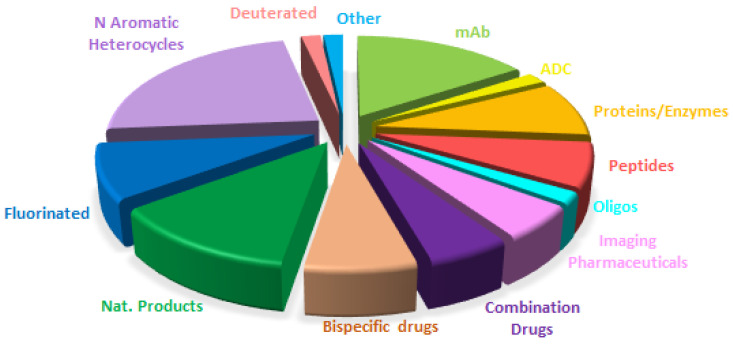
Similar to Figure 16 for 2022, taken with permission from ref. [9]. Copyright 2023, MDPI.

A comparison of the drugs approved this year (Figure 17) with those in 2023 (Figure 14) and 2022 (Figure 18) reveals that the distribution is very similar, with a clear consolidation of biologics and TIDES in detriment to small molecules.

Oncology, rare diseases, dermatology, infectious diseases, and contrast drugs are the main indications for the drugs approved by the FDA in 2024. In the small molecules class, kinase inhibitors are also the most frequent mode of action.

We confer the honorific title of “2024 Drug of the Year” to Resmetirom (Rezdiffra^TM^) because it is the first specific treatment for non-cirrhotic, non-alcoholic steatohepatitis (NASH), a disease that typically requires a liver transplant in late stages.

The pharmaceutical industry continues to demonstrate strong growth, with heavy investments in drug delivery systems, medical devices, and especially artificial intelligence (AI). The latter holds immense potential and uncertainty—what impact will it have on drug discovery? This will undoubtedly be revealed in the coming years.

Another pressing question for analysts regards the impact of political crises, new administrations, and shifting geopolitics on drug discovery.

As we bring this annual report to an end, we must highlight the rising cost of drugs, particularly those for chronic diseases. This implies that many of these groundbreaking treatments are available to only a small portion of the population. As the Access to Medicine Foundation has pointed out, the pharmaceutical industry continues to fall short in providing access to new drugs in low- and middle-income countries [10].

## Figures and Tables

**Table 1:** Biologics approved by the FDA in 2024 [1].

Trade Name ^a^	Active Ingredient ^a^	Class	Indication
Alhemo^TM^	Concizumab-mtci	mAb	Hemophilia A and B
Anktiva^TM^	Nogapendekin alfa & inbakicept	Protein combination	Bladder cancer
Bizengri^TM^	Zenocutuzumab-zbco	mAb	Non-small cell lung cancer and pancreatic Adenocarcinoma
Ebglyss^TM^	Lebrikizumab-lbkz	mAb	Atopic dermatitis (eczema)
Hympavzi^TM^	Marstacimab-hncq	mAb	Hemophilia A And B
Imdelltr^TM^	Tarlatamab-dlle	mAb	Extensive-stage small cell lung cancer
Kisunla^TM^	Donanemab-azbt	mAb	Alzheimer’s disease
Letybo^TM^	LetibotulinumtoxinA-wlbg	Protein	Improve the appearance of moderate-to-severe glabellar lines
Nemluvio^TM^	Nemolizumab-ilto	mAb	Prurigo nodularis (itchy firm lumps on the skin)
Niktimvo^TM^	Axatilimab-csfr	mAb	Chronic graft-versus-host disease
Piasky^TM^	Crovalimab-akkz	mAb	Paroxysmal nocturnal hemoglobinuria
Tevimbra^TM^	Tislelizumab-jsgr	mAb	Esophageal squamous cell carcinoma
Unloxcyt^TM^	Cosibelimab-ipdl	mAb	Cutaneous squamous-cell Carcinoma
Vyloy^TM^	Zolbetuximab-clzb	mAb	Gastric sr gastroesophageal adenocarcinoma
Winrevair^TM^	Sotatercept-csrk	Fusion protein	Pulmonary arterial hypertension
Ziihera^TM^	Zanidatamab-hrii	mAb	HER2-positive biliary tract cancer

^a^ Trade name used in the U.S.

## Data Availability

Not applicable.
